# PPARs as Key Transcription Regulators at the Crossroads of Metabolism and Inflammation

**DOI:** 10.3390/ijms25084467

**Published:** 2024-04-18

**Authors:** Manuel Vázquez-Carrera, Walter Wahli

**Affiliations:** 1 Department of Pharmacology, Toxicology and Therapeutic Chemistry, Faculty of Pharmacy and Food Sciences, University of Barcelona, 08028 Barcelona, Spain; 2 Institute of Biomedicine of the University of Barcelona (IBUB), University of Barcelona, 08028 Barcelona, Spain; 3 Spanish Biomedical Research Center in Diabetes and Associated Metabolic Diseases (CIBERDEM)-Instituto de Salud Carlos III, 28029 Madrid, Spain; 4 Pediatric Research Institute-Hospital Sant Joan de Déu, 08950 Esplugues de Llobregat, Spain; 5 Lee Kong Chian School of Medicine, Nanyang Technological University Singapore, Singapore 308232, Singapore; 6 Center for Integrative Genomics, University of Lausanne, CH-1015 Lausanne, Switzerland; 7 Toxalim, INRAE UMR 1331, F-31300 Toulouse, France

The metabolic and immune systems are complex networks of organs, cells, and proteins that are involved in the extraction of energy from food; this is to run complex cellular processes and defend the body against infections while protecting its own tissues, respectively. Metabolism and immunity are fundamental to the survival of an organism, from its conception and throughout its entire lifespan. They are evolutionarily conserved and interdependent, with their regulation deeply commingled and consolidated. The disruption of these finely tuned regulatory mechanisms can lead to the development of metabolic diseases, in which inflammation plays a critical role [1]. High plasticity is a major characteristic of metabolism and immunity. This plasticity is largely governed by gene expression changes that are orchestrated by sophisticated interactive mechanisms responding to extra- and intra-cellular signals, transcription factor activation or deactivation, epigenetic modifications, and post-transcriptional and post-translational modifications of newly transcribed RNAs and proteins [2,3,4].

In the past 20 years, the crucial role of transcription factors has become increasingly evident and well documented for both the metabolic and immune systems. In particular, some of their dysfunctions have been associated with the development of metabolic diseases. In this context, members of the nuclear hormone receptor superfamily, which comprises 48, 49, and 47 receptors in humans, mice, and rats, respectively [5], have been extensively studied due to their ligand-dependent activity; this makes them extremely interesting targets for the development of therapeutic interventions using synthetic compounds [6,7,8,9]. Three of these receptors, the peroxisome proliferator-activated receptors α, β/δ, and γ (PPARα, PPARβ/δ, and PPARγ, respectively), are eminent regulators of energy metabolism and inflammation. Most of their positive gene regulatory actions are mediated by PPAR:retinoid X receptor (RXR) heterodimers that bind short DNA motifs called peroxisome proliferator response elements, which are found in the regulatory regions of their target genes. Interestingly, ligand-activated PPARs can also downregulate gene expression via negative protein–protein interactions with proinflammatory transcription factors such as nuclear factor-κB (NF-κB), signal transducer and activator of transcription (STAT), and activator protein-1 (AP-1). The transrepressive effects of the PPARs outlined here participate in their anti-inflammatory activities [10].

Natural ligands, such as fatty acids and many of their derivatives obtained from food or produced by triglyceride lipolysis and de novo lipogenesis, activate PPARs by binding to their ligand-binding pocket [11,12]. Examples of the synthetic PPAR agonists used in clinical practice include fibrates and glitazones, which activate mainly PPARα and PPARγ, respectively. Isotype-based compound screening has identified single-, dual-, and pan-PPAR agonists such as pemafibrate (PPARα), elafibranor (PPARα-PPARβ/δ), saroglitazar (PPARα-PPARγ), and chiglitazar and lanifibranor (PPARα-PPARβ/δ-PPARγ), which are currently at different stages of drug development for various conditions [11,13]. Below, we highlight a few selected findings that illustrate the roles of each of the three PPAR isotypes in metabolic diseases and inflammation (Figure 1).

PPARα is abundantly expressed in organs with high levels of fatty acid oxidation, such as the liver, brown adipose tissue, heart, proximal tubules of the kidneys, and intestinal mucosa. It is well known for its role in energy homeostasis, and particularly for its critical function in the adaptive response to fasting, in which the liver plays a key role. In this process, PPARα is involved in all aspects of lipid metabolism. Mice with the hepatocyte-specific deletion of *Ppara* have impaired ketone body production in response to fasting, and develop metabolic dysfunction-associated steatotic liver disease (MASLD, formerly referred to as non-alcoholic fatty liver disease [NAFLD]) and hypercholesterolaemia [14]. Interestingly, the expression of the xenobiotic nuclear receptor constitutive androstane receptor (CAR) is stimulated by PPARα. When drug-activated in mice, CAR works as a negative regulator of PPARα by competing for the PPARγ coactivator 1 α (PGC1α), which downregulates PPARα-mediated lipid metabolism [15]. On the contrary, the activation of PPARα with the hypolipidaemic drug fenofibrate leads to increased fatty acid metabolism, resulting in profound changes in the hepatic fatty acid profile and endocannabinoid-related mediators that may further promote a drop in hepatic lipids, feed efficiency, and, consequently, body weight [16]. In light of recent results, the exploration of PPARα as a therapeutic target in diseases associated with lipid metabolism disorders continues to garner great interest [17]. Interestingly, a plethora of natural products that activate PPARs has also drawn attention; this includes herpetrione, a natural lignan isolated from *Herpetospermum caudigerum*. Herpetrione binds to and activates PPARα, thereby exerting various hepatoprotective effects; for example, it alleviates metabolic dysfunction-associated steatohepatitis (MASH) when hepatic fat build-up causes inflammation (hepatitis) and scarring in diet-induced liver disease models [18]. In addition to its metabolic roles, PPARα also regulates several hepatokines with autocrine and endocrine functions, such as fibroblast growth factor 21 (FGF21). Furthermore, as PPARα controls inflammation-modulating pathways, the use of PPARα agonists has been tested for the treatment of steatohepatitis in humans. The beneficial effects of PPARα have also been observed in other inflammatory conditions such as age-related inflammation, inflammatory skin and bowel diseases, and inflammatory osteoarthritis [10,19].

PPARβ/δ plays an important role in the liver, skeletal and heart muscles, skin, gut, placenta, adipose tissue, and brain. PPARβ/δ, like PPARα, participates in the control of carbohydrate and lipid metabolism in the liver. In MAFLD, PPARβ/δ reduces lipogenesis, alleviates inflammation and endoplasmic reticulum (ER) stress, ameliorates insulin resistance, and decreases the expression of several inflammatory cytokines [20,21]. In skeletal muscle cells, PPARβ/δ prevents ER stress-associated inflammation and insulin resistance through an adenosine monophosphate-activated protein kinase (AMPK)-dependent mechanism [22]. PPARβ/δ maintains slow oxidative fibers, at least in part by increasing the expression of PGC1α at the transcriptional level. Furthermore, the absence of PPARβ/δ in mouse skeletal muscle promotes obesity and diabetes [23]. Due to these beneficial effects, PPARβ/δ has been suggested as a possible therapeutic target for metabolic syndrome. From a more global point of view, PPARβ/δ has a broad expression pattern in almost all tissues, where it is involved in several functions other than those mentioned above [24]. For instance, PPARβ/δ ligands regulate the oxidative status and inflammatory response in an inflamed corpus luteum, which is of interest in the context of health problems that can be caused by inflammation in the female reproductive system, such as infertility [25]. Multiple studies have addressed the dual function of PPARβ/δ in cancer, which is most likely influenced by genomic and epigenomic alterations and modifications during tumor development. However, its involvement in cancer growth must be studied further, as a potent “hallmark” role of PPARβ/δ in tumor angiogenesis, cancer progression, and metastasis has been described [26].

PPARγ is expressed at high levels in adipose tissue, the colon, and macrophages. Polyunsaturated fatty acids, oxidized and nitrated fatty acids, 15-HETE, 9/13-HODEs, 13-oxo-ODE, 15-deoxy-D12,14-prostaglandin J2, and oxLDL components are PPARγ ligands. Two isoforms of PPARγ, which differ at their N termini, show divergent expression patterns. The shorter PPARγ1 is found in many organs and tissues including the gut, brain, vascular cells, and immune and inflammatory cells. PPARγ2 is mostly found in adipose tissue and the intestines. PPARγ is a key regulator of adipocyte differentiation and promotes fatty acid storage, glucose metabolism, and anti-inflammatory M2 macrophage activation. PPARγ is the target of glitazones and promotes insulin sensitivity by augmenting the uptake of fatty acids and the release of adiponectin by adipocytes [27]. Activated PPARγ also inhibits the growth of cancer cell lines. The overexpression of TRIB3, a member of the Tribbles family of pseudokinases, blunts the antiproliferative effect of PPARγ ligands in breast cancer cells via the H3K4 trimethylation of the PPARG locus, which reduces PPARγ expression. Accordingly, the expression levels of TRIB3 may predict the efficacy of PPARγ ligand treatment in breast cancer [28]. Activated PPARγ has exhibited some potential in the treatment of central nervous system (CNS) diseases in preclinical models. However, clinical trials in amyotrophic lateral sclerosis, Parkinson’s disease, and Alzheimer’s disease have produced disappointing results, possibly due to the insufficient exposure of the brain to the tested PPARγ ligands. More recently, a novel blood–brain barrier (BBB)-penetrating PPARγ agonist, leriglitazone, exhibited potential in the treatment of CNS diseases [29]. PPARγ can also ameliorate macrovascular and microvascular lesions in atherosclerosis and reduce the risk of cardiovascular disease [30]. The systemic inactivation of PPARγ has remained unexplored for some time since the whole-body invalidation of *Pparg* leads to embryonic lethality in mice. A model that maintains PPARγ expression in the placenta, but with whole-body *Pparg* deletion, was recently developed. These mice are completely deprived of adipose tissue and present a complex phenotype that is characterized by organomegaly, severe type 2 diabetes mellitus, and metabolic inflexibility [31]. Using this model, it was shown that PPARγ controls ectopic adipogenesis and cross-talks with myogenesis during skeletal muscle regeneration [32]. The anti-inflammatory roles of PPARγ in intestinal epithelial cells, which regulate mucosal immune responses and prevent inflammatory bowel disease, have already been reviewed elsewhere [12].

This brief non-exhaustive review of the roles of PPARs in metabolism and inflammation underscores the multifaceted activity of these receptors, thereby highlighting the challenge of obtaining a comprehensive understanding of their functions and regulation in healthy and diseased whole organisms. The impressive diversity of PPAR functions is paralleled by the broad variety of natural compounds that are currently known to act as PPAR activators. The occurrence of these molecules and their combinations in various tissues depend on the physiological conditions (e.g., nutrition, exercise, and circadian rhythms) and pathophysiological conditions (e.g., chronic low-grade inflammation and the cluster of abnormalities associated with metabolic syndrome, cancer, and obesity). In addition to the activators, the levels and combinations of the three PPARs in a cell nucleus, which are difficult to study, together with the components of the transcriptional machinery (coactivators, corepressors, and the preinitiation complex) eventually determine the final gene responses that affect the physiology of the whole organism, including the organ–organ dialogues that are essential to PPAR regulation. Furthermore, host–microbiota interactions not only influence PPAR responses, but are also impacted by them [33]. In this context, the reciprocal interaction between PPARα and the intestinal microbiota has been recently reviewed [34]. Microbiota-derived compounds modify PPARα signalling, while PPARα activation impacts the profile, diversity, and viability of the intestinal microbiota. Furthermore, PPARα affects processes connected to metabolism, immunity, immunological tolerance, and gut permeability. The rapidly progressing unveiling of PPAR functions and the regulation of PPAR activity by natural and synthetic compounds in whole-body physiology should aid in the design of enhanced drugs and novel therapeutic interventions.

## Figures and Tables

**Figure 1:**
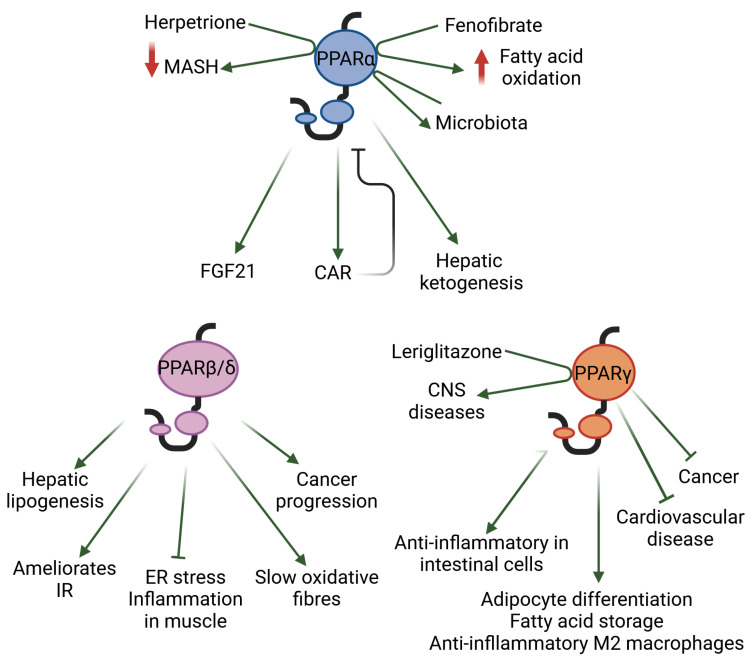
The selected roles, as discussed herein, of each of the three PPAR isotypes in metabolic diseases and inflammation are shown. CAR: constitutive androstane receptor; CNS: central nervous system; ER: endoplasmic reticulum; FGF21: fibroblast growth factor 21; IR: insulin resistance; MASH: metabolic dysfunction-associated steatohepatitis.
